# Is menopause still evolving? Evidence from a longitudinal study of multiethnic populations and its relevance to women’s health

**DOI:** 10.1186/s12905-020-00932-8

**Published:** 2020-04-19

**Authors:** Shirley Chan, Alyssa Gomes, Rama Shankar Singh

**Affiliations:** Department of Biology, McMaster University, Hamilton, Ontario Canada

**Keywords:** Mate choice, Menopause, Perimenopause, Sex-hormones, Menstruation, Fertility

## Abstract

**Background:**

To reflect on the impact of changing patterns of delayed marriage and reproduction and to seek evidence as to whether menopause is still evolving, characteristics of the menopause transition were investigated within and between ethnic populations in this study.

**Methods:**

A cross-sectional analysis was conducted using data on 747 middle-aged women obtained from the Study of Women’s Health Across the Nation (SWAN) from 1996 to 2008. The ethnic groups included: Afro-American, Chinese, Japanese, Caucasian, and Hispanic. Perimenopause age and duration, menopause age, and hormonal indicators of menopause were examined across five ethnicities.

**Results:**

We found a similar window of menopause age within populations, but no significant difference in perimenopause and menopause age between populations. The rate of increase of follicle-stimulating hormone and testosterone differed significantly in Hispanics and African-Americans during the menopause transition period.

**Conclusions:**

The broad window of variation in age at menopause within the population and the absence of significant differences between populations, in combination with population variation in menopause symptoms, suggest that menopause is a relatively recently evolved and still evolving trait. Under the mate choice theory of menopause, menopause is the result of the accumulation of infertility mutations in older women due to men’s preference for younger mates. We propose a *shifting mate choice-shifting menopause* model which posits that, as the age of mate choice/marriage shifts to older ages, so will the age at menopause, and that menopause is a transient phase of female fertility; it can de-evolve, be delayed, if not disappear completely. Integrated longitudinal menopausal studies linked with genomics and hormonal studies on diverse ethnic populations can provide valuable information bearing on women’s health and personalized medicine.

## Background

**Fig. 1:**
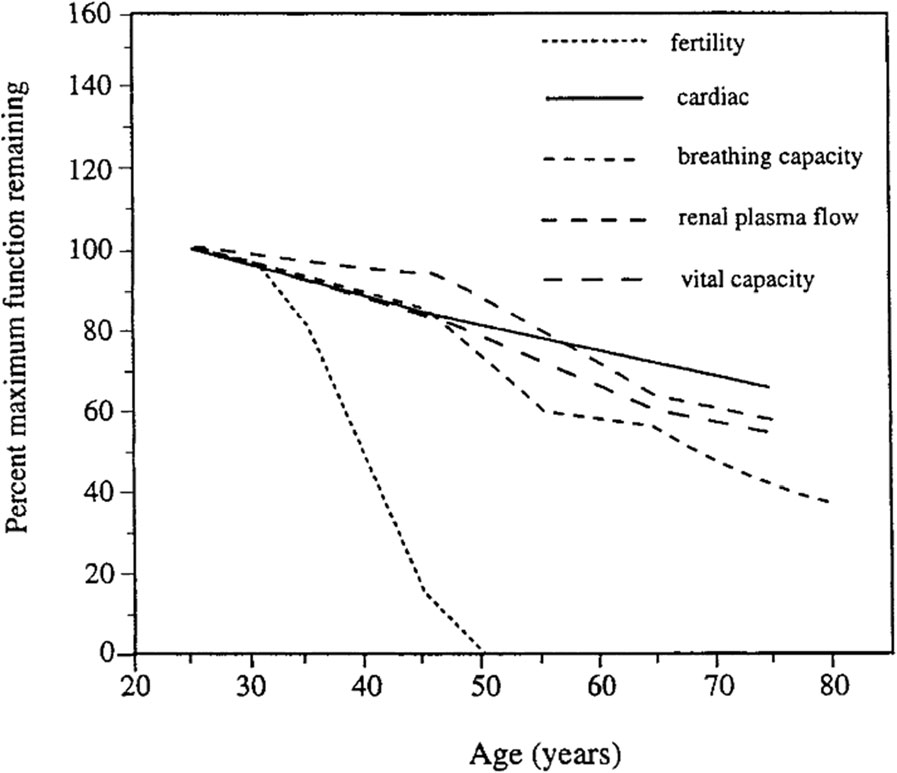
Comparative senescence of physiological functions in human females. Taken from Peccei, 2001; redrawn from Hill and Hurtado, 1991 [3, 4]

Life-history traits are considered important in the evolution of species; one example is the evolution of life history that causes humans to age. The basic concept stems from the disproportion of young individuals in a population as the result of age-associated increases in extrinsic mortality [1, 2]. Selective forces acting on young individuals are greater, allowing beneficial early-life alleles to be selected for even if they are detrimental in later life [1, 2]. This intrinsic mortality manifests as a decline in all body systems followed by progressive deterioration of physiological functions as individuals age (Fig. 1) [3, 4]. While reproductive ability also declines with age, reproductive senescence (aging) is unique in that it follows an accelerated decline relative to other physiological functions [3, 5–7]. Termination of reproductive function is observed at roughly 50 years of age yet, in the absence of this accelerated decline, it could be extended to 70 years of age [3, 5–7]. Furthermore, this rapid decline in reproductive ability is found uniquely in females; male reproductive senescence patterns follow the same gradual decline shown in other physiological functions [3, 5–7].

Accelerated reproductive decline in females conflicts with classic evolutionary theory^2^. Reproduction is the mechanism allowing for the transfer of genes that promote survival and thus is crucial to organismal fitness. To cease reproduction early, which releases selection for genes promoting survival, is non-adaptive as, from an evolutionary perspective, being fertile until death would, ceteris paribus, maximize organismal fitness. This is the life history strategy observed in most animal populations, including chimpanzees and other primates, the closest living relatives to humans [2, 8]. Despite this seemingly contradictory nature of early reproductive decline, menopause (reproductive cessation with post-reproductive lifespan) is universal in all women.

Studies involving menopause revolve around two perspectives: a medical perspective with a view to understanding post-menopause women’s health [9], and an evolutionary perspective with a view to understanding its origin and evolution [10]. Evolutionary research on menopause revolves around the forces contributing to the evolution of the menopause trait in female reproductive history, which are broadly summarized into adaptive and non-adaptive approaches. The adaptive view examines selective pressures favoring early reproductive decline to increase fitness, such as increased maternal investment or trade-off from grandparenting, in the evolution of menopause [11, 12]. The non-adaptive approach does not view menopause as the direct result of selection. Menopause is considered an artifact revealed by the relatively recent increase in lifespan or the result of antagonistic pleiotropy, where early fertility is favored at the cost of late fertility [3, 11]. More recently, using a population genetics approach, menopause was proposed as the non-adaptive outcome of the accumulation of infertility mutations rendered neutral due to male preference for younger women and, as a result, the exclusion of older women from reproduction [10]. While the debate centers on the origin of menopause, there is a pertinent need to understand when menopause was introduced over the course of human evolution and whether it is still evolving in terms of its pre- and post- menopausal effects on fitness and health.

Approximations for the timing of the appearance of menopause can be suggested based on the theories of the origin of menopause. The Grandmother Theory posits that grandmothers assist with raising their grandchildren to improve their survival and reproductive success, rather than continuing reproduction to older ages, which would pose an increased risk to the mother [3]. This strategy would increase fitness, making survival to older ages advantageous and increase selection for late-life alleles, which ultimately slows the aging of physiological processes. In general, slower aging comes at a cost of reduced fertility at younger ages [12, 13]. The appearance of delayed maturity, a representation of delayed aging, was estimated at 1.5 million years ago, which suggests that menopause is an old trait [3, 12, 14]. Further evidence from cross-species analysis with chimpanzees revealed that follicular number, a marker of menopausal status, has been conserved over the course of human evolution [8, 15, 16]. Similarities in the age at reproductive cessation and primordial follicular patterns between chimpanzees and humans suggest that menopause can be considered a fixed trait of ape evolution [8, 15, 16]. While circumstantial evidence from anthropological data implies that menopause originated as early as the appearance of our ancestor *Homo erectus* 1.8 million years ago, it remains uncertain as to whether menopause presented itself during that era [3, 17, 18].

There is, however, a growing body of research that may provide compelling evidence for menopause being a relatively recent evolutionary trait. Due to the distinct menopausal experiences among women, research for ethnic-specific menopause syndromes has become a topic of interest. Since menopause has a genetic basis, ethnic differences in menopause phenotype may reflect ethnic differences in their genome pertaining to menopause. While all human genomes can be traced back to an ancient African ancestor, only 8% of genetic variation between individuals differentiates populations [19]. Hence, if the menopause phenotype varies across ethnicities, it provides evidence of menopause as a recently evolved trait within the last 5000 to 50,000 years, after humans migrated out of Africa and settled into different regions across continents. While past studies have claimed a universal menopausal syndrome, current findings support a link between ethnicity and health indicators pertaining to menopause [20–25]. Among menopausal vasomotor symptoms, Western women report higher rates of hot flushes than Asian women, who report muscle pain more often [21, 24]. Among hormonal values, rates of AMH decline, which reflect ovarian reserve, were found to be lower in African-Americans compared to white women [23, 25]. Disease risks associated with menopause were also found to vary with ethnicity [23]. With these observations in mind, we have proposed a population genetics theory for the evolution of menopause [10]. Here, we consider a second, follow-up, question: Is menopause still evolving and if so, can it de-evolve, i.e. can it be delayed? To answer this question, we need to know the nature of variation in menopause within and between populations and infer if menopause is an actively evolving trait.

The objective of this study was to investigate if menopause is a fixed or a dynamic and still-evolving trait. Understanding ethnic patterns in menopausal experiences is crucial to determine when menopause evolved, yet research lacks a wide range of ethnic groups, focusing solely on Caucasian/white women and African-American women. Important reproductive stages such as perimenopause (irregular menstruation) are also not extensively studied. To accomplish our objective, we used data from a long-term American study of multi-ethnic populations to analyze population-ethnic differences in the age of perimenopause onset and the associated hormonal changes that occur during the menopause transition.

## Methods

### Sample population

The data used for this study were obtained from the American organization the Study of Women’s Heath Across the Nation (SWAN). SWAN is a longitudinal, multi-ethnic, multi-site study of middle-aged women. Data on the women enrolled in SWAN, from their baseline visit in 1996–1997 up to the tenth follow-up visit, were accessed through the publicly available datasets on ICPSR. The design of SWAN was previously published by Sowers et al. [26]. The ongoing SWAN studies collect data annually either through interviewer or self-administered questionnaires across seven clinical sites in the USA, including California, Los Angeles, Detroit, Chicago, Pittsburgh, Boston, and Newark. Participants were self-identified as Black or African-American, Non-Hispanic Caucasian, Chinese, Japanese, and Hispanic (Central American, Cuban or Cuban American, Dominican, Mexican or Mexican American, Puerto Rican, South American, Spanish or other Hispanic). Eligibility into the baseline group required being 42–52 years of age with an intact uterus, at least one intact ovary, non-use of exogenous hormones during the prior 3 months, and reported a menstrual period within the last 3 months.

SWAN’s baseline study in 1996–1997 consisted of 3302 pre- and perimenopausal women, of which there were 1550 Caucasians (46%), 935 African Americans (28%), 286 Hispanics (9%), 250 Chinese (8%), and 281 Japanese (9%) women. Our study used data only from the premenopausal women from the baseline collection up to their tenth visit (1996–2008), which decreased the sample size to 1727 women. The sample was further narrowed to 1242 women by excluding premenopausal women or women with no data by the end of the tenth visit. We also examined their responses for the variable OOPHORE, which tracked whether a woman underwent oophorectomy procedures, the surgical removal of one or both ovaries. Women who underwent oophorectomy procedures, which were coded as 2 (“yes”) prior to perimenopause were removed, decreasing the sample size to 1151 women. Also excluded from the sample were women that lacked data the year before perimenopause was diagnosed and women with inconsistent menstruation patterns (i.e. were declared menopausal for more than 1 year, then had normal menstruation), resulting in a final sample size of 747 women. The purpose of this data scrutiny and reappraisal was to make sure that we had suitable, comparable data from all ethnic populations and that women had attended regular yearly visits for 10 years and all had completed the tenth visit.

### Menopause transition stage

In SWAN, menopausal status was declared based on a woman’s menstrual bleeding pattern over the past 12 months. Women were categorized as premenopausal, perimenopausal, or menopausal. “Premenopause” referred to regular monthly menstrual cycles with menstrual cycles occurring in the past 3 months; “perimenopause” referred to irregular menstrual cycles with menstrual bleeding in the last 12 months but not in the last 3 months; “menopause” referred to no menstruation for a minimum period of 12 months. For each yearly visitation, SWAN questionnaires collected menstrual characteristics. Each woman was asked if they had bled in the past 3 months and 12 months, which were assigned the variable BLD3M and BLDNG respectively. Responses were coded as 1 (“no”) or 2 (“yes”) in the database. Premenopausal status was designated when 2 was coded for both BLD3M and BLDNG; perimenopausal when 2 was coded for BLDNG and 1 was coded for BLD3M; and menopausal when 1 was coded for both BLD3M and BLDNG.

In this study, we determined the perimenopause age of onset, menopause age of onset, and the duration of perimenopause for each individual in the sample. A woman’s age at perimenopause onset was determined using their age at which they transitioned from premenopausal to perimenopausal status. Similarly, menopausal age of onset was the age at which they transitioned from premenopausal or perimenopausal to menopausal. The duration of perimenopause was calculated from the difference between menopausal age of onset and perimenopausal age of onset. Cases in which a woman transitioned from premenopausal to menopausal within a year (i.e. perimenopause duration was < 1 year) were referred to as “sudden menopause”. To determine the variation in perimenopause onset and menopausal age across ethnic groups, the respective mean and median ages were calculated for each ethnic group. To determine the variation in the duration of perimenopause, the mean duration was also calculated for each ethnic group, excluding women with sudden menopause.

### Variation in hormone concentration

In SWAN, morning fasting blood was drawn annually on days 2–7 of the menstrual cycle (follicular phase) for regularly cycling women. For women that stopped menstruating or lacked a follicular phase sample, a fasting blood sample was drawn within 90 days of the follow-up visit date. Each sample was assayed for the hormones dehydroepiandrosterone sulphate (DHAS), sex-hormone binding globulin (SHBG), follicle-stimulating hormone (FSH), testosterone (T), and estradiol (E2), conducted at the RSP CLASS Laboratory at the University of Michigan. DHAS and T were measured with the automated CLASS laboratory modified ACS: 180-based chemiluminescent assay developed in the CLASS laboratory. SHBG and FSH were measured with a two-site chemiluminescent assay. E2 concentrations were measured using the estradiol-6 III immunoassay performed on the ADVIA Centaur instrument. Results are listed in the SWAN database under the variables DHAS, T, SHBG, FSH, and E2AVE, respectively.

For this paper, we examined the DHAS, SHBG, FSH, T, and E2 concentrations for each individual in the study in two time frames: the year prior to perimenopause onset and the year at menopause onset. The year of perimenopause onset was not used due to the fluctuations and high variability of hormone concentrations during perimenopause. Using these hormone concentration values from the two time frames, the absolute and relative (percentage) change in hormone concentration over the menopause transition was calculated separately for each individual. Within each ethnic group, the mean DHAS, SHBG, FSH, T, and E2 concentrations were calculated for both time frames to assess how endogenous reproductive hormone concentrations compare among ethnicities. The mean absolute and relative change in hormone concentrations over the menopause transition for each ethnic group was also calculated to analyze how the rates of increase in endogenous reproductive hormone concentrations over the menopause transition compare among ethnicities.

### Statistical analysis

All variables examined in this study were compared across all ethnicities for statistical significance of differences using single-factor ANOVA and t-tests with two samples, assuming equal variances as needed in Microsoft Excel software. Results with *p* < 0.05 were considered to reflect statistically significant differences. Multivariate analyses of sociodemographic and life style variables in SWAN data were done by Gold et al. [9] and were not performed in this study.

Source of data: SWAN Study Repository: https://www.icpsr.umich.edu/icpsrweb/ICPSR/series/00253

Source of methodology: Sowers et al. [26].

## Results

**Fig. 2:**
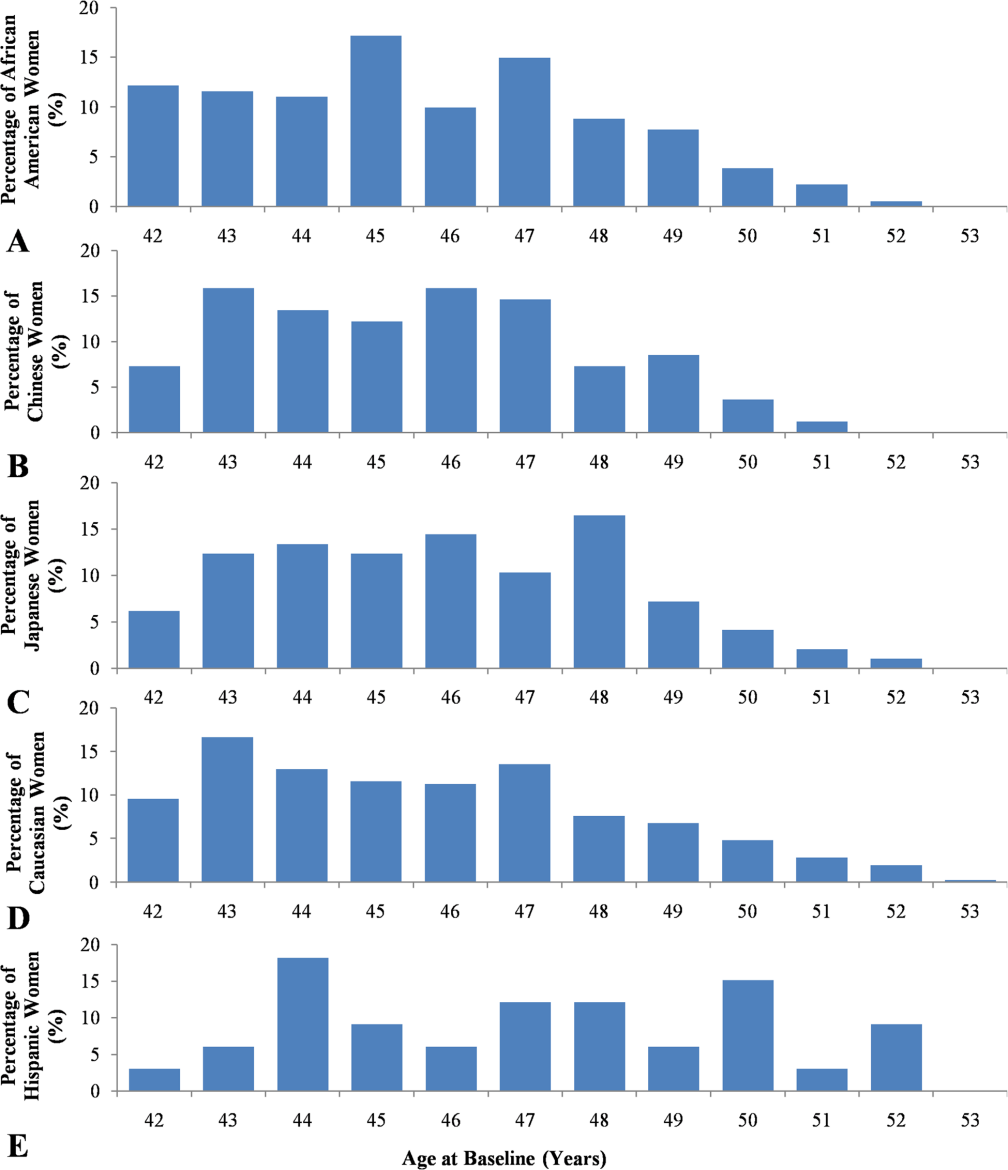
Ethnic-specific variation in women’s age (years) of perimenopause and menopause onset by race/ethnicity. **a** Perimenopause onset distribution. Cumulative percentage of the number of people in each ethnic group that began perimenopause as a function of a women’s age (years), using data obtained from SWAN Baseline to Visit 10. **b** Menopause onset distribution. Cumulative percentage of the number of people in each ethnic group that started menopause as a function of a women’s age (years), using data obtained from SWAN Baseline to Visit 10

Data from a total of 747 SWAN participants were used in this study. The number of participant varied from a low of 33 in Hispanic to a high of 354 in Caucasian (Table 1). At baseline, all women were premenopausal (Fig. 2) and by the tenth follow up visit, were either perimenopausal or menopausal. A significant number of women, ranging from a low of 34% in the Japanese population to a high of 51.5% in Hispanic subjects, experienced sudden menopause, i.e. went into menopause within a year, while all other women underwent perimenopause (Table 1). Of the latter, a significant portion (ranging from 24.2% in Hispanic subjects to 56.7% in Japanese subjects) finished perimenopause and entered menopause by the tenth visit. A significant number were still in perimenopause at the tenth visit (Table 1).

**Table 1:** Distribution of SWAN participants by race/ethnicity based on menopause status

Ethnicity	Total number of participants	Women with Sudden Menopause	Women with Perimenopause	
	n (%)	n (%)	Ongoing (%)	Finished (%)
African-American	181 (24.2)	73 (40.3)	25 (13.8)	83 (45.9)
Chinese, CA	82 (11.0)	31 (37.8)	5 (6.1)	46 (56.1)
Japanese, JA	97 (13.0)	33 (34.0)	9 (9.3)	55 (56.7)
Caucasian, NHW	354 (47.2)	134 (37.9)	55 (15.5)	165 (46.6)
Hispanic	33 (4.4)	17 (51.5)	8 (24.2)	8 (24.2)

Abbreviations: *SWAN* Study of Women’s Health across the Nation; *CA* Chinese-American; *JA* Japanese-American; *NHW* Non-Hispanic Whites

### Onset age of perimenopause and menopause

**Fig. 3:**
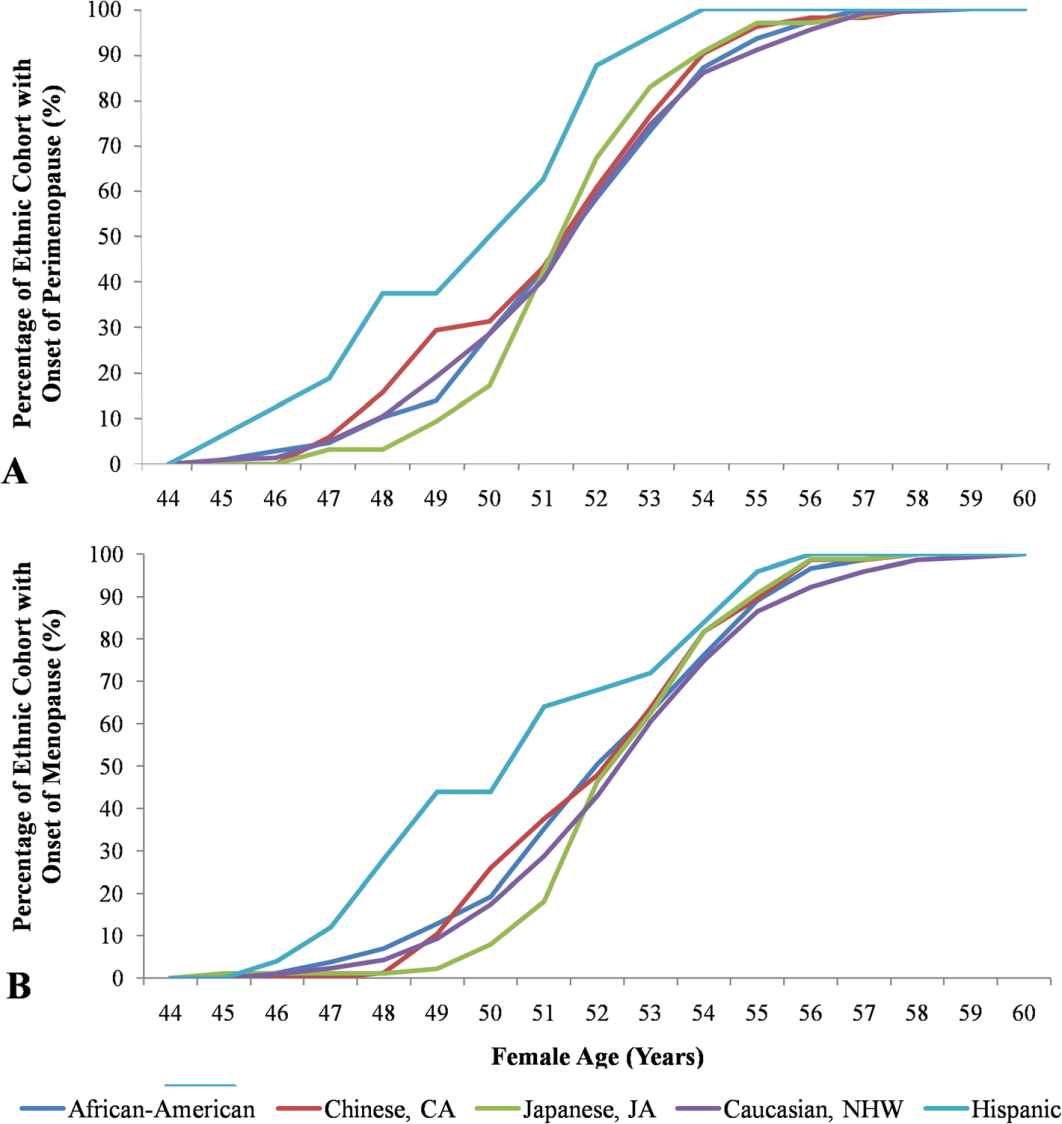
Age distribution of participating women by race/ethnicity at SWAN Baseline. Percentages of (**a**) African-American women, **b** Chinese and Chinese-American women, **c** Japanese and Japanese-American women, **d** Caucasian and non-Hispanic White women, and **e** Hispanic women

The overall average age of perimenopause onset across the entire sample was 51.79 ± 2.51 years (Fig. 3). The mean (± SD) age of perimenopause was almost identical among population with the exception of Hispanic which was lower by 2 years, but not significantly so (Table 2). The overall average age of menopause across the entire sample was 52.63 ± 2.48 years. There were no differences in menopause age between populations, except in Hispanics where the mean age of menopause was lower by 2 years (50.84 years).

**Table 2:** Mean age (years) at perimenopause and menopause onset, and duration of menopause transition

Ethnicity	Average Age at Baseline (Yr.) (Range)	Average Age at Perimenopause Onset (Yr ± SEM) (Range)	Average Age at Menopause (Yr ± SEM) (Range)	Average Duration of MT (Yr ± SEM) (Range)
African American	45.6 (42–51)	51.87 ± 0.24 (45–58)	52.46 ± 0.20 (46–58)	1.34 ± 0.07 (1–4)
Chinese, CA	45.6 (42–51)	51.55 ± 0.36 (47–58)	52.44 ± 0.26 (48–58)	1.35 ± 0.08 (1–3)
Japanese, JA	46.0 (42–52)	51.92 ± 0.25 (45–58)	52.88 ± 0.20 (45–58)	1.33 ± 0.08 (1–3)
Caucasian, NHW	45.7 (42–53)	51.90 ± 0.18 (45–60)	52.85 ± 0.15 (46–60)	1.27 ± 0.04 (1–4)
Hispanic	47.1 (42–52)	49.94 ± 0.66* (45–56)	50.84 ± 0.60* (46–56)	1.12 ± 0.13 (1–2)

Abbreviations: *SWAN* Study of Women’s Health Across the Nation; *CA* Chinese-American; *JA* Japanese-American; *NHW* Non-Hispanic Whites; *Yr* year; *SD* standard deviation; *MT* menopause transition. Values are **from SWAN baseline to visit 10.** * represents significant difference (*P* < 0.05). For sample size see Table 1

The Hispanic population showed significant differences in the average age of perimenopause and menopause, but there was considerable heterogeneity in Hispanic samples originating from different Central American countries (Fig. 2). The significant differences in perimenopausal and menopausal age in the Hispanic population may be due to the slightly older age of the women participating in this study (Table 1, Fig. 3). No significant differences were observed for either perimenopause age or menopause age between all other populations (*P* = 0.83 and 0.26, respectively). Japanese women experienced perimenopause and menopause the latest among the ethnic group, although the difference was again found to be non-significant.

**Fig. 4:**
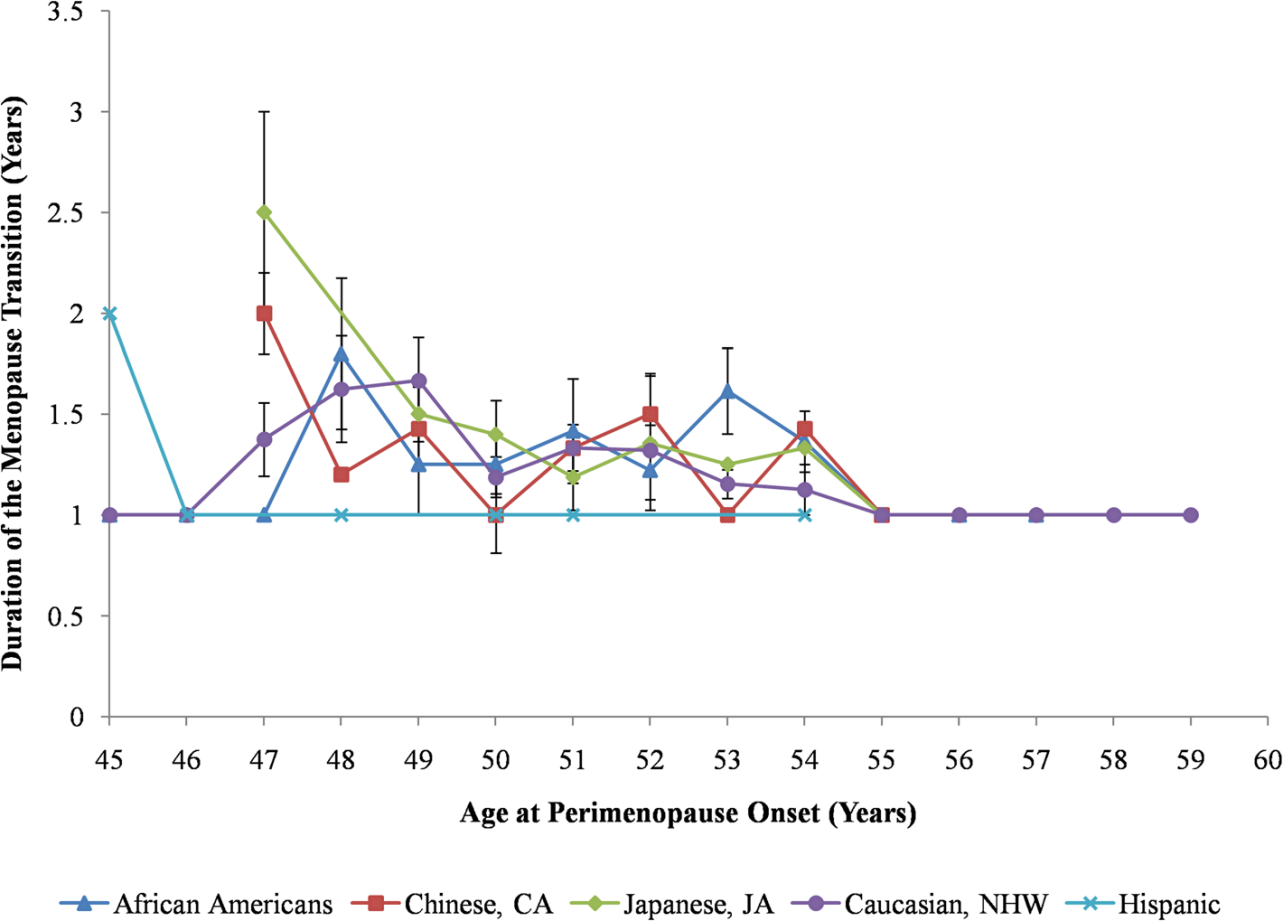
Average duration of perimenopause as a function of perimenopause age of onset by race/ethnicity. Duration was obtained from age of onset of perimenopause and menopause using data from SWAN Baseline to Visit 10. Data shown as a mean ± SEM of women in each ethnic group

Interestingly, while there is a wide window around the mean age of menopause, the average length of perimenopause, in years, across the entire sample was rather small: 1.30 ± 0.58 years. What this means is that, once a woman goes into perimenopause, she can expect to transition into full menopause within one to 2 years. Differences in the length of perimenopause were statistically not significant across ethnicities (*P* = 0.76), lasting approximately 1.3 years (Table 2). The overall duration of perimenopause decreased as women approached the age of 50 years (Fig. 4).

### Serum hormone concentrations

**Fig. 5:**
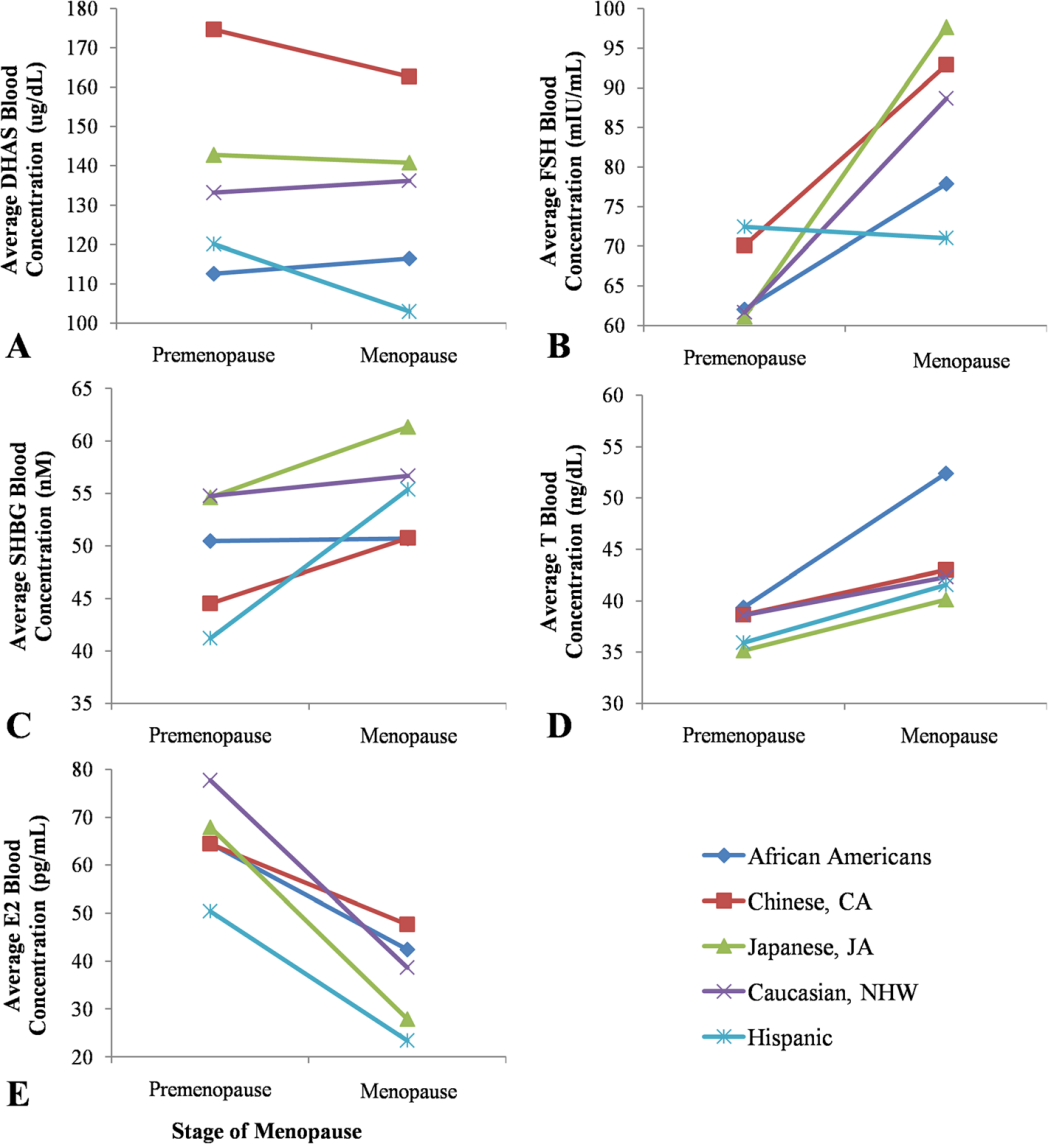
Ethnic specific variation in hormone levels associated with the menopause transition by race/ethnicity. Values shown as the mean of women in each ethnic cohort for the year prior to perimenopause onset (considered “premenopause”) and the year of menopause onset. Blood values for (**a**) DHAS. ANOVA indicated a significant difference in premenopausal and menopausal DHAS levels across ethnicities (*P* = 3.7E-7 and *P* = 9.3E-4, respectively). **b** FSH. ANOVA indicated a significant difference in menopausal FSH levels across ethnicities (*P* = 0.0099), while premenopausal FSH levels did not differ significantly (*P* = 0.52). **c** SHBG. ANOVA indicated a significant difference in premenopausal SHBG levels across ethnicities (*P* = 0.03), while menopausal SHBG levels did not differ significantly (*P* = 0.09). **d** T. ANOVA indicated a significant difference in menopausal T levels across ethnicities (*P* = 0.09), while premenopausal T levels did not differ significantly (*P* = 0.51). **e** E2.ANOVA determined no significant difference in E2 values was found between ethnicities in both premenopausal and menopausal women (*P* = 0.4, 0.25 respectively)

Relative and absolute levels of hormones are shown in Table 3 and plotted in Fig. 5. Before going to individual hormones, some general patterns were noticeable. Firstly, populations appeared to harbor varying levels of premenopausal and menopausal levels of hormones: DHAS was significantly different for both pre- and menopausal women, FSH showed significant difference only for menopausal (and not premenopausal), SHBG has significant difference only for premenopausal women (and not menopausal), T was significantly different for only menopausal (not pre), and E2 showed no significant differences in either premenopausal or menopausal women. Secondly, populations varied in the ranking of their hormone levels, meaning that no population has consistently high or low levels for all hormones. Finally, in general, for any given hormone, the levels seemed to increase or decrease similarly among populations, which suggests that relative changes may be more important than the absolute amount of a given hormone.

**Table 3:** Mean absolute and relative (percent) increase in reproductive hormone values over the menopause transition by race/ethnicity

Ethnicity	African Americans	Chinese, CA	Japanese, JA	Caucasian, NHW	Hispanic
**DHAS (ug/dL)**	0.47 (−92.8–178.8)	−13.80 (−188.3–201.9)	− 0.94 (− 149.1–129.6)	1.24 (−170.9–227.6)	−23.21 (− 200.5–80.7)
**DHAS (%)**	8.55 (−68.7–444.8)	4.85 (− 81.6–604.7)	8.66 (− 64.7–291.7)	9.86 (− 82.4–250.9)	0.20 (− 78.9–130.8)
**FSH (mIU/mL)**	27.86* (−93.0–181.8)	29.51* (− 102.8–188.7)	44.91* (− 47.6–225.3)	39.61* (− 78.7–185.3)	5.39* (−26.3–79.4)
**FSH (%)**	157.77 (−84.0–1986.1)	145.00 (− 72.4–1238.0)	224.19 (−65.8–5364.3)	184.59 (−89.1–2404.7)	114.75 (−54.9–680.0)
**SHBG (nM)**	1.63 (− 109.6–149.9)	7.33 (− 98.8–145.9)	9.08 (− 117.6–108.9)	4.81 (− 97.2–137.5)	6.45 (− 34.9–38.4)
**SHBG (%)**	51.51 (−76.9–3848.0)	53.10 (− 76.77–782.14)	37.31 (− 68.8–414.7)	26.88 (− 81.4–670.7)	21.15 (− 38.0–107.6)
**T (ng/dL)**	15.97* (−27.7–226.8)	3.00* (− 35.3–62.1)	4.10* (− 45.4–67.0)	3.54* (− 52.5–129.6)	18.89* (− 14.8–81.4)
**T (%)**	61.51* (−87.1–704.4)	9.81* (− 79.2–149.4)	24.57* (− 59.0–259.1)	26.24* (− 85.7–664.2)	113.83* (− 52.9–767.9)
**E2 (pg/mL)**	− 29.29 (− 483.2–240.0)	−21.16 (353.8–1158.3)	−35.94 (− 723.8–549.6)	−45.42 (− 531.0–195.4)	−40.38 (− 340.0–22.2)
**E2 (%)**	29.05 (− 94.9–3000.6)	25.12 (− 97.6–1421.5)	−0.54 (− 99.2–2224.9)	−7.24 (− 98.05–962.56)	−4.77 (− 94.5–223.6)

Abbreviations: *SWAN* Study of Women’s Health Across the Nation; *DHAS* dehydroepiandrosteronesulphate; *FSH* follicle-stimulating hormone; *SHBG* sex-hormone binding globulin; *T* testosterone; *E2* estradiol; *CA* Chinese-American; *JA* Japanese-American; *NHW* Non-Hispanic Whites. The values are from SWAN baseline to visit 10; values in parentheses are range: the first dash means a decrease in the level of the hormone; the second dash is for range. * represents significant difference (*P* < 0.05); For sample size see Table 1

Across all ethnicities, absolute and relative (percent) amount of FSH, SHBG, and T levels increased when transitioning from premenopausal to the menopausal stage (Table 3, Fig. 5). Relative changes in the levels of DHAS tended to increase for all ethnicities, whereas absolute increases in DHAS were observed for only African-Americans and Caucasians. Absolute E2 changes tended to decrease across all ethnicities but, relatively, E2 only decreased in Japanese, Caucasian, and Hispanic women. Relative and absolute changes in serum DHAS, SHBG, and E2 concentrations did not differ significantly across ethnicities. Relative increases in serum FSH levels did not differ significantly across ethnicities (*P* = 0.70), but the absolute increase differed significantly across ethnicities (*P* = 0.01). Absolute increases in serum FSH levels in African-American and Hispanic women were significantly lower than in Japanese and Caucasian women. Relative and absolute increases in serum T levels also differed significantly across ethnicities (*P* = 1.1E-4, 5.45E-5). African-Americans and Hispanic women had significantly greater increases in relative and absolute T concentrations than Caucasians, Chinese, and Japanese women.

## Discussion

Insight into the association between ethnic diversity and the characteristics of a woman’s menopause transition may provide evidence for the timing of the appearance of menopause in human evolution. In this study, we evaluated perimenopause and menopause age, perimenopause duration, and the associated reproductive hormonal changes in a longitudinal, multi-ethnic sample from the cross-sectional study SWAN [9]. While the results of this study revealed lack of a consistent ethnicity-specific difference in the timing of the menopause transition, there were significant changes in the associated hormones which, together with variations within populations in the menopause “window” and population-specific variation in menopausal symptoms, are suggestive of menopause as a recently evolved and still evolving trait in human evolution.

Before discussing the significance of these results, it is important to state the rationale for this study. From the women’s health point of view, it is important to know if menopause is still evolving and, if so, can it de-evolve, i.e. can it be delayed or even eliminated. To be able answer this question, we need to know the nature of variation in menopause. The SWAN study (and many others reviewed by Gold et al. [9]) looked into the effect of lifestyle factors on menopause. Controlling for sociodemographic, lifestyle, and health factors affecting the final menstrual period (FMP), Gold et al. [9] found that “racial/ethnic groups did not differ in age at the FMP. Higher educational level, prior oral contraceptive use, and higher weight at baseline, as well as being employed, not smoking, consuming alcohol, having less physical activity, and having better self-rated health over follow-up, were significantly associated with later age at the FMP”. These results suggest that menopause is a complex trait, but it is interesting to note that, in spite of the effect of socioeconomic factors, the onset-age “window” of menopause remains more or less stable from population to population (Fig. 2). Our reduced data set was designed to increase the chances of discovering ethnic differences in the onset age of menopause; we found it to vary significantly in Hispanic population only. In the following, we review our findings and use them to propose a model of “shifting mate choice-shifting menopause” and predict that there is population variation in the onset age of perimenopause and that the shifting patterns of mate choice and marriage will lead to a shift, a delay in the onset age of menopause.

### Ethnic variation in perimenopause age

At present, this is the first assessment of the age of perimenopause onset in a multi-ethnic sample. This study has identified a significantly earlier perimenopause onset in women of Hispanic descent compared to women of Caucasian, African-American, Chinese, and Japanese descent. Hispanics experience perimenopause approximately 2 years earlier, which suggests that changes in the reproductive systems are still taking place and that menopause has not stopped evolving separately in each population. While this result may be suspect because of small sample size and heterogeneity among samples originating from different Central American countries, as we show below, other studies have reported similar results.

Previous perimenopause literature is highly limited. Mentions of perimenopause age in other literature are either not quantified or they refer to the mean age of women in the perimenopause cohort, rather than onset age. Of the feasible literature, McKinlay et al. [27] has reported the inception of perimenopause to be 47.5 years, earlier than determined in our study (51.8 years). It should be noted that McKinlay et al.’s [27] perimenopause age was not specific to any ethnicity and was limited to women in Massachusetts. Sammel et al. [28] also examined the menopause transition across ethnicities, albeit limited to Caucasians and African-Americans. The specific age was not listed, but it was found that African-American women entered perimenopause earlier than Caucasians, a finding that was not observed in this study [28]. Differences in the reported age in McKinlay et al.’s [27] study and the variation in African-American and Caucasian women perimenopause age of onset in Sammel et al.’s [28] study can be attributed to definition bias introduced in our study. Other studies define perimenopause onset using early perimenopause (menstrual irregularity). In contrast, we used late perimenopause (3 to 11 months of amenorrhea) due to a change in the questionnaire following the 1997–1999 follow-up (SWAN), which led to overestimation of perimenopause onset age. It is likely that a change in perimenopause definition will yield results similar to other studies, as our results are consistent with Sammel et al.’s [28] finding that entry into late perimenopause did not differ significantly between African-American and Caucasian women. Although it is probable that the definition of perimenopause influenced observed trends, it is likely that an ethnic variation in perimenopausal age exists. Perimenopause age in Indian women was reported to be 44.69 years, which is prominently earlier than any reported perimenopause age for US women as a whole and thus suggestive of differential biological timing of perimenopause present among populations [29]. More studies with diverse population samples are needed.

### Ethnic variation in menopause age

In agreement with wide variation in menopause age across women worldwide, heterogeneity in menopausal age among ethnicities was found only in Hispanics in our study. Hispanic women experienced menopause 2 years earlier than women of other ethnicities, which mimics the results found for perimenopause age. Again, this result may due to small sample size, but several studies have reported a 2 year difference in menopausal age between Hispanic and Caucasian women [30–32]. Age of menopause in Hispanic populations has been reported at 48.5 years for 15 countries across Latin America and 47.9 years in Mexican women, whereas Caucasian populations experience menopause at 50 years [33, 34]. Despite the higher menopausal age recorded in this study (51 vs. 53 years; Fig. 2), which was attributed to differences in methodology, our findings support previous literature. Furthermore, we suggest this difference is unlikely to be the result of lifestyle factors such as smoking prevalence, which accelerates menopause by 1–2 years [27, 32, 35]. Previous analysis of SWAN participants have shown that smoking frequency was lower among Hispanics than Caucasians and African Americans, which was consistent with other studies [36, 37]. Hence, the earlier onset in Hispanic women may be due to a different biological clock for the timing of menopause onset compared to other ethnicities.

Studies of candidate gene polymorphisms affecting menopausal age have found that the rs16991615 SNP in *MCM8* locus, a gene involved in the repair of double-stranded DNA breaks reduces age at menopause by 2.3 years for Hispanic women [38]. The samers16991615 SNP exerts the opposite result in Caucasian women, increasing menopausal age by 0.82 years [39]. The effect of an SNP may vary according to ethnicity, which suggests that menopause may have co-opted different genes in ancestral Hispanic populations.

Whether a difference in menopausal age between African-Americans and Caucasians exists is unclear. Bromberger et al. [35] proposed that African-American women experience menopause 6 months to a year earlier than Caucasian women. This seems plausible given that studies of African women, specifically from Ghana and Nigeria, determined that menopause occurred at 48–49 years, which is earlier than Caucasian women [30, 32, 34]. In contrast, several studies did not report differences in menopausal age between Caucasians and African-Americans [28, 30, 31]. Our findings support the latter, as both the mean and median menopausal age in both ethnicities was similar. It is possible that the small African American sample size (*n* = 19) and high percentage of smokers could have contributed to Bromberger et al.’s [35] observations.

The similarity in menopausal age observed in both African-American and Caucasian women led us to expect similar evolution of menopause in their ancestral populations. Interestingly, however, many of the Caucasian associated SNPs studied in candidate gene polymorphisms associated with menopause age were poorly replicated or not replicable in African-Americans in GWAS studies, which suggest that African-Americans are not as similar to Caucasians as initially believed [39, 40]. Given the high linkage disequilibrium, African-Americans were suggested to have a variant of the SNP at the associated loci, although it was not identified [39, 40]. If variants exist in African-Americans, it is possible that the effect of the variant would be similar to the Caucasian SNP to result in the similar phenotype (i.e. menopause age) observed between these two ethnicities. This seems plausible since the SNP findings were able to be replicated in African Americans, such as the rs365132 SNP at the *UIMC1* locus, with effects similar to Caucasians (i.e. increased menopausal age by ~ 0.4 years) [40]. Further research is required to determine the variation between Caucasians and African-Americans, as SNPs associated with menopause onset in African-Americans have not been well-studied.

Menopausal age for Chinese women from China and Singapore and Japanese women from Japan was roughly 50 years in both populations, which resembles the Caucasian menopausal age [34, 41–44]. Concurrent with our results, similarity in menopausal age between Caucasian, Chinese, and Japanese women have been reported in the literature [42, 45–47]. Several SNPs associated with menopausal age in Caucasians were replicated to the same effect and direction in Chinese women from Shanghai, suggesting that there are some shared menopausal evolutionary traits between these two ethnicities [48].

Recent studies have suggested that Japanese women entered menopause at later ages than Caucasian women [28, 30, 31, 37]. While the mean menopausal age did not differ significantly between Caucasian and Japanese women in our study, we did observe a higher percentage of Japanese women entering perimenopause and menopause later than other ethnicities, which supports past findings [28, 30, 31, 37]. If differences in menopause age exist between the two ethnicities, this may indicate differences in menopause affecting loci. One study reported several SNPs associated with menopause age in Caucasians could not be replicated in Japanese women in GWAS studies; however this was a very small study [39]. Further research is needed to determine whether or not Caucasian SNPs can actually be replicated in Japanese women or whether other variants exist.

### Ethnic variation in the duration length of perimenopause

This is also the first study reporting the duration of perimenopause among different ethnic groups. In spite of the heterogeneity in the timing of the menopause transition, our findings show that the length of perimenopause was consistent across all ethnicities (Fig. 4). The duration of perimenopause appears negatively correlated with the age of menopause. This result presents the possibility that the intrinsic mechanisms underlying the progressive loss in ovarian function from the perimenopause to menopause stage is shared among individuals. Combined with the data for timing of the menopause transition, it is hypothesized that the timing of onset is what separates the menopause phenotype between ethnicities, as opposed to the duration. Future research can be directed to understanding why and how the timing of menopause has been shifted in different populations over the course of human evolution to result in the current ethnic variation in the onset of the menopause transition. One evolutionary theory explores the idea that women of ancestral populations with lower female biased dispersal would experience earlier menopause, although the required information about ancestral ecologies in these populations is lacking [49].

McKinlay et al. [27] determined the length of perimenopause to be 3.7 years, which was markedly higher than the 1.3 years reported in this study. Again, this can be partly attributed to the definition bias of perimenopause, which would have led to an underestimation of the perimenopause duration in this study (Fig. 4). Alternatively, the methodological differences used to determine the duration was also a factor. For example, McKinlay et al. [27] determined duration by subtracting the mean age of menopause from perimenopause onset. This could lead to under/overestimation of the duration since it was not representative of the duration in individuals. There is individual variation in duration of perimenopause; hence, the duration should be determined by averaging the duration in each individual, which was done in this study. Future studies in perimenopause length are encouraged to average individual duration for greater accuracy of the duration. Additionally, further research is needed to determine if the similarity in duration in ethnic groups still exists when early perimenopause is used to define perimenopause onset, as opposed to late perimenopause.

### Ethnic variation in hormone concentrations

Across all ethnicities, E2 concentrations declined over the menopause transition, as expected. As the menopause transition progresses, there is progressive loss of ovarian follicles, which produces E2, leading to overall reduced E2 levels [32]. Consistent with studies conducted by Randolph et al. [50] and Kim et al. [51], mean E2 levels did not differ significantly across ethnicities. Similarities between E2 levels across ethnicities may be linked to earlier observations of the perimenopause duration. Klaiber et al. [52] noted that women with lower E2 levels had shorter menopause transition duration. Since all ethnicities were similar in E2 levels, the duration was not expected to differ between ethnicities, which are consistent with our results.

Since changes in E2 are correlated to changes in FSH, we did not expect differences in serum FSH across ethnicities either. However, we found a significantly lower FSH increase in Hispanics and African-Americans compared to other ethnicities during the menopause transition. These ethnic differences in FSH levels were thought to be linked to ethnic variation in pituitary-ovarian feedback during the menopause transition, such as variation in estrogen sensitivity [53–55]. Greater sensitivity to estrogen decline is associated with higher FSH levels, as estrogen is a negative regulator of FSH. Examination of polymorphisms in *ESR1*, which codes for an estrogen receptor, has shown that the rs2234693 polymorphism was significantly associated with Caucasian women menopause onset, although further research is needed for other ethnicities [56]. In contrast, other studies have reported higher FSH levels in Hispanics and African-Americans, regardless of menopausal status [50, 53]. This was attributed to the lack of adjusted BMI hormonal values in our study and different hormonal collection times. Fundamentally, both our results and Randolph et al.’s [50, 53] have consistently noted an ethnic difference in FSH levels.

Similar to FSH levels, there was an ethnic difference in the increase of T levels across the menopause transition. Premenopausal T concentrations did not vary among ethnicities, but in menopausal women, T was significantly higher in Hispanic and African-American women compared to other ethnicities, suggestive of ethnic differences in T metabolism over the menopause transition. Literature to support this observation is scarce because T assays have difficulties reporting the low T concentrations in women, making it challenging to study in menopausal women [57]. A study by Luckey et al.’s [58] provides a possible indicator of ethnic differences in T metabolism by examining rates of bone loss during the menopause transition, as lower T levels are suggested to contribute to greater bone loss. In Luckey et al.’s [58] study, African-American women were found to have lower rates of bone loss than white women, suggestive of higher T levels in African-Americans, which is consistent with our findings in this study. Other studies have found lower T levels for African-Americans and Hispanics compared to other ethnicities, which was attributed to BMI adjusted values [50, 51]. Overall, we can affirm that ethnic variation for T levels exists.

Several studies have correlated changes in SHBG to changes in T, which may suggest that ethnic differences in T could be attributed to ethnic differences in SHBG during the menopause transition [59, 60]. This was unlikely given that ethnic differences in SHBG in menopausal women and SHBG rate of increase over the menopause transition were not observed, which was consistent with findings from Burger et al. [59]. Alternatively, studies have found associations between DHAS and T, which may also suggest ethnic differences in DHAS as a factor for ethnic differences in T [60, 61]. While ethnic differences were noted in DHAS levels in both premenopausal women and menopausal women, no ethnic differences were found in the rate of DHAS increase. Similar findings have also been reported in other studies [59]. It thus seems that ethnic differences in DHAS changes are not specifically associated with the menopause transition, and is unlikely to be the cause of ethnic differences in T during the menopause transition, so other factors are likely responsible for the ethnic differences in T changes. One possibility involves androstenediol, which is secreted similarly to DHAS during the menopause transition [62]. Its concentrations are thought to affect the balance of androgens over the menopause transition, hence research determining if ethnic differences exist for androstenediol, along with other possibilities for the ethnic differences in T, would be beneficial [62].

As mentioned before, there are several limitations to this study. The first is regarding the participants. The age of participants was limited to 42–55 years, which may have biased the perimenopause and menopause age to older ages. This may not be a significant limitation, given that, in a study done by Luborsky et al. [37], they reported ethnic variation in premature menopause (menopause age < 40 years) consistent with our study. The exclusion of women with inconsistent bleeding patterns in this study could also have biased the results. Although not shown, we retested our findings with these exceptions added, but overall the findings remained the same. Secondly, late perimenopause was used to define perimenopause onset, leading to an overestimation of perimenopause age, underestimation of menopause duration, and irregularity in premenopausal hormone values. Additionally, use of BMI adjusted hormonal values could have altered our findings, as BMI has been shown to affect hormonal values significantly, positively for T and negatively for all other tested hormonal values [53]. It is also possible that hormone therapy could have interfered with our findings; however, as explored by Kim et al. [51], hormone therapy does not deviate from the results comparing ethnicities; rather, it only reduces the differences between groups.

### A “shifting mate choice-shifting menopause” model of female fertility

The main results regarding variation in age of menopause, from this study as well as others, can be summarized as follows. Firstly, all human populations, regardless of socioeconomic status, show a roughly 15-year window of menopause (45–60 Yr), which means that there is plenty of variation within populations. Secondly, many lifestyle factors are known to affect the age of menopause, but with the exception of premature menopause, the menopause window persists in all populations. Thirdly, there are no significant ethnic differences in the onset age of perimenopause, presumably because there has not been sufficient time since the origin of menopause to produce such differences. Furthermore, as we show below, the absence of variation among populations may indicate the role of similar mate choice mechanisms (i.e. male preference for younger mates) operating in all human populations. Fourth, there are significant changes in the levels of associated hormones within as well as between populations. Finally, different populations show ethnicity/race-specific menopause symptoms; for example, vasomotor symptoms were more common in Afro-American and Hispanic women than in other ethnicities [63]. This suggests a role of both biological and cultural factors. We take these results to mean that menopause is a relatively recent trait, and most variation in the trait is present within populations with relatively little variation between populations. This is not surprising in view of what is known about human genetic variation in general [19].

We use these results to extend the mate choice theory of menopause [10], which holds that menopause is the result of men’s preference for younger mates, leading to the accumulation of deleterious mutations affecting fertility in women deprived of reproduction. We propose (1) that the present menopause window (roughly 45–60 years) is directly related to the “mate choice window” in the present-day human population (roughly 15–30 years); (2) that the lack of between-variation in perimenopause is the result of a similar mate choice system operating in all human populations; and (3) that changing patterns of mate choice/marriage will ultimately lead to a shift in the onset age of menopause. This shift will not occur immediately as the pressure from the shifting mate choice will only have its effect if women of premenopausal age will choose to engage in reproduction. Under the mate choice theory, menopause is a transient phase of male-imposed female fertility and it will be delayed with changing patterns of delayed mate choice/marriage and reproduction.

### Clinical significance

To allude to the clinical significance of this study we introduce Mayr’s concept of *proximal* and *ultimate causes* [64]. Clinical practices are based on proximal causes of the disease and proximal or immediate cure or relief to the patient. Evolutionary studies, as the present one, relate to the ultimate causes and while they may not help the clinicians or the patients in the short term, they provide an evolutionary framework for understanding the disease and the variable symptoms and treatment response between patients in the long term. Menopause is not a disease; it is an evolutionary consequence of reproductive behavior imposed by mate choice practices in the long past. Our study shows that menopause is not an invariable trait and the results from this study will provide basis to help design personalized treatment. Integrated studies involving longitudinal menopausal studies linked with genomics and hormonal studies with diverse ethnic populations can provide valuable information bearing on women’s health and personalized medicine.

## Conclusions

This study allows us to reach two conclusions. From evolutionary perspective, the substantial variation in age at menopause within populations and the modest variation in hormonal changes and menopause symptoms between populations, together with the intact retention of “non-ovulatory reproductive system” in menopausal women suggests that, evolutionarily speaking, menopause is a relatively recent trait and is still evolving. The mate choice theory of menopause [10] predicts that, as the age of mate choice and marriage shift upward, so will the age at menopause as mate choice and menopause are causally connected. Future research should focus on examining menopause phenotypes in other ethnicities and see if the evolution of menopause and associated genetic polymorphisms can be linked to the known historic-geographic patterns of human evolution.

From health perspective, there is ethnic variation in the menopause phenotype that is consistent with other studies, specifically in age at perimenopause and menopause, and in FSH and T hormonal changes. Data from this report supports the finding that at least two distinct categories for the menopause phenotype exist: (1) earlier timing of menopausal transition with lower FSH and higher T levels, associated with African-American and Hispanic women, and (2) later timing of menopause transition with higher FSH and lower T levels, associated with Caucasian, Chinese, and Japanese women. In view of the fact that sex and reproduction-related genes have been shown to evolve quickly and that sex hormones provide a susceptible target of change, these menopause phenotypes open up the possibility of experimental manipulation of hormones as a personalized menopause therapy.

These results show potential for the continued evolution of menopause and possibly delayed appearance of menopause, allowing women more time for family planning.

## Data Availability

The data analyzed during the current study are available in the SWAN STUDY Repository: https://www.icpsr.umich.edu/icpsrweb/ICPSR/series/00253

## Abbreviations

AMH: Anti-mullerian hormone
ANOVA: Analysis of variance
BMI: Body mass index
CA: Chinese-American
DHAS: Dehydroepiandrosterone sulphate
E2: Estradiol
FMP: Final menstrual period
FSH: Follicle-stimulating hormone
GWAS: Genome-wide association studies
ICPSR: Inter-university Consortium for Political and Social Research
JA: Japanese-American
MT: Menopause transition
NHW: Non-Hispanic White
SD: Standard deviation
SEM: Standard error of mean
SHBG: Sex-hormone binding globulin
SNP: Single nucleotide polymorphisms
SWAN: Study of Women’s Health Across the Nation
T: Testosterone
US: United States
Yr: Year

## References

[1] Kirkwood TB, ASN. Why do we age? Nature. 2000;408(6809):233–8. 10.1038/35041682 11089980

[2] Croft DP, Brent LJ, Franks DA, Cant MA (2015). The evolution of prolonged life after reproduction. Trends Ecol Evol.

[3] Peccei JS (2001). Menopause: adaptation or epiphenomenon?. Evol Anthropol.

[4] Hill K, Hurtado AM. The evolution of premature reproductive senescence and menopause in human females, an evaluation of the “grandmother hypothesis”. Hum Nat. 1991;2(4):313–50. 10.1007/BF02692196 24222339

[5] Wood JW (1990). Fertility in anthropological populations. Annu Rev Anthropol.

[6] Faddy MJ, Gosden RG, Gougeon A, Richardson SJ, Nelson JF (1992). Accelerated disappearance of ovarian follicles in mid-life: implications for forecasting menopause. Hum Reprod.

[7] Austad SN (1994). Menopause: an evolutionary perspective. Exp Gerontol.

[8] Emery-Thompson M, Jones JH, Pusey AE, Brewer-Marsden S, Goodall J, Marsden D (2007). Aging and fertility patterns in wild chimpanzees provide insights into the evolution of menopause. Curr Biol.

[9] Gold EB, Crawford SL, Avis NE, Crandall CJ, Matthews KA, Waetjen LE (2013). Factors related to age at natural menopause: longitudinal analyses from SWAN. Am J Epidemiology.

[10] Morton RA, Stone JR, Singh RS (2013). Mate choice and the origin of menopause. PLoS Comput Biol.

[11] Pollycove R, Naftolin F, Simon JA (2011). The evolutionary origin and significance of menopause. Menopause..

[12] Hawkes K, O’Connell JF, Blurton Jones NG, Alvarez H, Charnov EL (1998). Grandmothering, menopause, and the evolution of human life histories. Proc Natl Acad Sci U S A.

[13] Kirkwood TB, Rose MR (1991). Evolution of senescence: late survival sacrificed for reproduction. Philos trans R Soc Lond B biol. Sci..

[14] Smith BH, Leakey R, Walker A (1993). The physiological age of KNM-WT 15000. The Nariokotome *Homo erectus* skeleton.

[15] Wood JW, O’Conner KA, Holman DJ, Brindle E (2000). The evolution of menopause by antagonistic pleiotropy. Homo..

[16] Cloutier CT, Coxworth JE, Hawkes K (2015). Age-related decline in ovarian follicle stocks differ between chimpanzees (*Pan troglodytes*) and humans. Age (Dordr).

[17] Smith BH (1991). Dental development and the evolution of life history in Hominidae. Am J Phys Anthropol.

[18] Trinkaus E (2011). Late Pleistocene adult mortality patterns and modern human establishment. Proc Natl Acad Sci U S A.

[19] Lewontin RC (1972). The apportionment of human diversity. Evol Biol.

[20] Avis NE, Stellato R, Crawford S, Bromberger S, Ganz P, Cain V (2001). Is there a menopausal syndrome? Menopausal status and symptoms across racial/ethnic groups. Soc Sci Med.

[21] Singh A, Kaur S, Walia I (2002). A historical perspective on menopause and menopausal age. Bull Indian Inst Hist Med Hyderabad.

[22] Avis NE, Brockwell S, Clovin A (2005). A universal menopausal syndrome?. Am J Med.

[23] Butts SF, Seifer DB (2010). Racial and ethnic differences in reproductive potential across the life cycle. Fertil Steril.

[24] I’m E, Koi Y, Chee W (2014). Ethnic differences in the clusters of menopausal symptoms. Health Care Women Int.

[25] Seifer DB, Golub ET, Lambert-Messalina G, Benning L, Anastasi K, Watts DH (2009). Variations in serum malaria inhibiting substance between white, black, and Hispanic women. Fertil Steril.

[26] Sowers MF, Crawford SL, Lobo RA, Kelsey J, Marcus R (2000). SWAN MD: a multicenter, multiethnic, community-based cohort study of women and the menopausal transition. Menopause: biology and pathobiology.

[27] McKinlay SM, Brambilla DJ, Posner JG (1992). The normal menopause transition. Maturitas..

[28] Sammel MD, Freeman EW, Liu Z, Lin H, Guo W. Factors that influence entry into stages if the menopausal transition. Menopause. 2009;16(6):1218–27. 10.1097/gme.0b013e3181a8f62b PMC2783664 19512950

[29] Ahuja M (2016). Age of menopause and determinants of menopause age: a PAN India survey by IMS. J Midlife Health.

[30] Gold EB, Bromberger J, Crawford S, Samuels S, Greendale GA, Harlow SD (2001). Factors associated with age at natural menopause in a multiethnic sample of midlife women. Am J Epidemiol.

[31] Henderson KD, Bernstein HL, Henderson B, Kolonel L, Pike MC (2008). Predictors of the timing of natural menopause in the multiethnic cohort study. Am J Epidemiol.

[32] Gold EB (2011). The timing of the age at which natural menopause occurs. Obstet Gynecol Clin N Am.

[33] Legorreta D, Montaño JA, Hernández I, Salinas C, Hernández-Bueno JA. AMEC research committee. Age at menopause, motives for consultation and symptoms reported by 40-59-year-old Mexican women. Climacteric. 2013;16(4):417–25. 10.3109/13697137.2012.696288 22888911

[34] Schoenaker DA, Jackson CA, Rowlands JV, Mishra GD (2014). Socioeconomic position, lifestyle factors and age at natural menopause, a systematic review and meta-analyses of studies across six continents. Int J Epidemiol.

[35] Bromberger JT, Matthews KA, Kuller LH, Wing RR, Meilahn EN, Plantinga P (1997). Prospective study of the determinants of age at menopause. Am J Epidemiol.

[36] Pérez-Stable EJ, Ramirez A, Villareal R, Talavera GA, Trapido E, Suarez L (2001). Cigarette smoking behavior among US Latino men and women from different countries of origin. Am J Public Health.

[37] Luborsky JL, Meyer P, Sowers MF, Gold EB, Santoro N (2003). Premature menopause in a multi-ethnic population study of the menopause transition. Hum Reprod.

[38] Chen CT, Fernández-Rhodes L, Brzyski RG, Carlson CS, Chen Z, Heiss G (2012). Replication of loci influencing ages at menarche and menopause in Hispanic women: the Women’s health initiative SHARE study. Hum Mol Genet.

[39] Carty C, Spencer K, Setiawan V, Fernandez-Rhodes L, Malinowski J, Buyske S (2013). Replication of genetic loci for ages at menarche and menopause in the multi-ethnic population architecture using genomics and epidemiology (PAGE) study. Hum Reprod.

[40] Chen CT, Liu CT, Chen GK, Andrews JS, Arnold AM, Dreyfus J (2014). Meta-analysis of loci associated with age at natural menopause in African-American women. Hum Mol Genet.

[41] Chang C, Chow SN, Hu Y (1995). Age of menopause of Chinese women in Taiwan. Int J Gynaecol Obstet.

[43] Akahoshi M, Soda M, Nakashima E, Tominaga T, Ichimaru S, Seto S (2002). The effects of body mass index on age at menopause. Int J Obes Relat Metab Disord.

[42] Tamada T, Iwasaki H (1995). Age at natural menopause in Japanese women. Nippon Sanka Fujinka Gakkai Zasshi.

[44] Loh FH, Khin LW, Saw SM, Lee JJ, Gu K (2005). The age of menopause and the menopause transition in a multiracial population, a nation-wide Singapore study. Maturitas..

[45] Goodman MJ, Grove JS, Gilbert F (1978). Age at menopause in relation to reproductive history in Japanese, Caucasian, Chinese and Hawaiian women living in Hawaii. J Gerontol.

[46] Boulet M (1990). The menopause and the climacteric in seven Asian countries. In: sixth international congress on the menopause.

[47] Boulet MJ, Oddens BJ, Lehert P, Vemer HM, Visser A (1994). Climacteric and *menopause* in seven south-east Asian countries. Maturitas..

[48] Shen C, Delahanty RJ, Gao YT, Lu W, Xiang YB, Zheng Y (2013). Evaluating GWAS-identified SNPs for age at natural menopause among Chinese women. PLoS One.

[49] Úbeda F, Ohtsuki H, Gardner A (2014). Ecology drives the intragenomic conflict over menopause. Ecol Lett.

[50] Randolph JF, Sowers M, Gold EB, Mohr BA, Luborsky J, Santoro N (2003). Reproductive hormones in the early menopausal transition: relationship to ethnicity, body size, and menopausal status. J Clin Endocrinol Metab.

[51] Kim C, Pi-Sunyer X, Barrett-Connor E, Stentz FB, Murphy MB, Kong S (2013). Sex hormone binding globulin and sex steroids among premenopausal women in the diabetes prevention program. J Clin Endocrinol Metab.

[52] Klaiber EL, Broverman DM, Vogel W, Peterson LG, Snyder MB (1997). Relationships of serum estradiol levels, menopausal duration, and mood during hormonal replacement therapy. Psychoneuroendocrinology..

[53] Randolph JF, Sowers M, Bondarenko IV, Harlow SD, Luborsky JL, Little RJ (2004). Change in estradiol and follicle-stimulating hormone across the early menopausal transition: effects of ethnicity and age. J Clin Endocrinol Metab.

[54] Weiss G, Skurnick JH, Goldsmith LT, Santoro NF, Park SJ (2004). Menopause and hypothalamic-pituitary sensitivity to estrogen. JAMA..

[55] Tepper PG, Randolph JF, McConnell DS, Crawford SL, El Khoudary SR, Joffe H (2012). Trajectory clustering of estradiol and follicle-stimulating hormone during the menopausal transition among women in the study of Women’s health across the nation (SWAN). J Clin Endocrinol Metab.

[56] Weel AE, Uitterlinden AG, Westendorp IC, Burger H, Schuit SC, Hofman A (1999). Estrogen receptor polymorphism predicts the onset of natural and surgical menopause. J Clin Endocrinol Metab.

[57] Miller KK, Rodner W, Lee H, Heir J, Sessile G, Shenfield D (2004). Measurement of free testosterone in normal women and women with androgen deficiency: comparison of methods. J Clin Endocrinol Metab.

[58] Luckey MM, Wallenstein S, Lapinski R, Meier DE (1996). A prospective study of bone loss in African-American and white women – a clinical research center study. J Clin Endocrinol Metab.

[59] Burger HG, Dudley EC, Cui J, Dennerstein L, Hopper JL (2000). A prospective longitudinal study of serum testosterone, dehydroepiandrosterone sulfate, and sex hormone-binding globulin levels through the menopause transition. Clin Endocrinol Metab.

[60] Morley JE, Perry HM (2003). Androgens and women at the menopause and beyond. J Gerontol A BiolSci Med Sci.

[61] Lasley BL, Santoro N, Randolf JF, Gold EB, Crawford S, Weiss G (2002). The relationship of circulating dehydroepiandrosterone, testosterone, and estradiol to stages of the menopausal transition and ethnicity. J Clin Endocrinol Metab.

[62] Lasley BL, Crawford S, McConnell DS (2011). Adrenal androgens and the menopausal transition. Obstet Gynecol Clin N Am.

[63] Green R, Santoro N (2009). Menopausal symptoms and ethnicity: the study of Women’s health across the nation. Women’s Health.

[64] Mayr E (1961). Cause and effect in biology. Science.

